# Collectanea

**Published:** 1827-07

**Authors:** 


### COLLECTANEA.

Floriferis ut apes in saltibus omnia libant,
Omnia nos, itidem, depascimur aurea dicta.

### PRACTICAL MEDICINE.

*Treatment of Intermittent Fever.*—In a paper by Dr. MACKINTOSH, on Bleeding in the Cold Stage of Intermittent Fever, (Edinburgh Medical and Surgical Journal, April,) we find the following observations:—

For some little time before the paroxysm begins, the patient complains of lassitude and debility, (but which is not actual weakness, but oppression,) with diminished circulation on the surface of the body, more particularly in the extremities; they are benumbed and feel cold; the sense of cold becomes universal, and a shivering takes place. The body soon becomes affected with universal tremors. There is great prostration of strength, confusion of mind, sometimes delirium, anxious and quick breathing, the patient being unable to take in a deep inspiration, and oppression at the præcordia; and the pulse, which is sometimes quick, at others slow, is often irregular, but always oppressed.

It is rare, avowedly, that complete coma, apoplexy, or convulsions, take place; but that these do occur, the records of medicine fully prove; and I have seen more than one person die in the cold stage. The convulsive tremors are not under the control of the will, and, if I can trust my own sensations, they affect internal as well as external muscles.

I attribute all these symptoms to congestion in internal organs. I imagine no one who has seen this disease will deny that there is sufficient evidence of the balance of the circulation being upset, and that an irregular determination of blood takes place. It appears to have quitted the surface, by the coldness and shrinking of the extremities. It is forced upon internal organs to a serious extent, impeding their functions.

Thus the convulsive tremors, confusion of mind, and pain in the head, are produced by congestion of the brain and spinal marrow.

The difficulty of breathing is owing principally to venous engorgement of the lungs, and the overloaded state of the right side of the heart. The spleen, liver, and mesenteric vessels, also suffer from congestion, and frequent attacks lead to structural derangements of these viscera. These pathological conclusions are fully established by comparing the symptoms with the morbid appearances found in the examination of the bodies of those who have expired in the cold stages, and are corroborated in a remarkable manner, by the good effects of opening a vein, when all the symptoms give way in an instant. I have seen men in the most severe sufferings, relieved after the abstraction of six, eight, and ten ounces of blood. I have known three ounces suffice, and on one or two occasions only I had to bleed to the extent of two pounds. The relief (which is the most perfect relief that can well be conceived) is so sudden when a good orifice can be made, that it has surprised and delighted every one who has seen my practice. I shall never forget the pleasure which was expressed by my excellent friend Dr. Kellie, who is well known to the professional world, when he first saw the practice carried into execution in my hospital. Before proceeding further, I may shortly mention the appearances which I have seen on opening the bodies of a few men who expired in the cold stage.

An immense quantity of black blood was found in the vessels of the head, the lungs, the heart and large vessels near it, the liver, spleen, and mesenteric vessels. The spleen (according to Sir John Pringle, and some of the older writers,) has sometimes been found ruptured from over-distention. So great a quantity of blood have I seen in such cases in the lungs, that large sections of them sank in water. These portions, when washed and deprived of blood by pressure, regained their natural appearance and buoyancy. The liver, when washed and squeezed, will also regain its natural colour.

This pathological condition of organs has been denominated "congestion." It simply implies that the balance between the arterial and venous systems is lost for the time, the latter being overloaded or congested, and not that the circulation in any organ, or set of organs, is entirely obstructed. This indeed does happen in those extreme cases in which reaction does not take place, and the individuals die in the cold stage.

From the time of Celsus, an idea has prevailed that death would be the inevitable consequence of bleeding in the cold stage. The late Dr. Gregory used to make this assertion in his lectures, and, to give it more weight, he quoted the expression of Celsus, "hominem jugulare." Other lecturers on the practice of physic make similar statements. It would be curious and interesting to know whether they have ever seen this melancholy consequence of bleeding in the cold stage; or whether they have been led to suppose it would be produced, assuming, as Cullen did, that the paroxysm is produced by debility, want of energy of the brain, and spasm of the mouths of the extreme vessels.

After numerous observations, made with the utmost anxiety to discover truth, and from the sensations I experienced during a protracted intermittent in a marshy country, I submitted myself to the experiment; in the first instance, disregarding altogether the force of authority upon this subject. Bark and all the usual remedies had failed, and my health was materially injured. A vein was opened in the arm, and, before twelve ounces were abstracted, the rigors ceased, and all its unpleasant accompaniments: there was no hot stage, no sweating stage; a pleasant sense of heat succeeded the painful one of cold, and, instead of weakness, I was sensible of an acquisition of strength. Afterwards I bled many other individuals, and always with the same results. This was done so long ago as the year 1810; but as I cannot appeal, at this distance of time, to living witnesses, I shall confine the statement I am now to make to the cases of intermittents which have occurred to me within these few years, and which have been seen by many witnesses who were well qualified to judge, from their experience and standing in the profession, and also by many of my pupils.

CASE I.—James Ward, admitted into Royal Ordnance Hospital, in November 1823.

Has suffered several attacks of intermittent annually, since the year 1809, when he served in the expedition to Walcheren. Of late his indispositions were long, and left him more and more debilitated. Several of my pupils watched this man closely every third day, for a number of attacks, with a view to bleed him in the cold stage, but they were not fortunate enough to arrive in time. They bled him twice, however, in the hot fit, from the severity of the symptoms, and with considerable temporary relief, but without preventing or mitigating the violence of the subsequent paroxysms. Some time afterwards, in the presence of Drs. Lucas and Robinson, two of my pupils, I bled him from a vein in the arm, during the cold stage: it was very severe; the rigors were violent, and the sense of coldness insupportable. He complained much of his head and loins; his face was of a livid colour, and the vessels of the conjunctiva turgid with blood. Pulse 100 or 105, and oppressed; breathing short and anxious, and, to use his own expression, he felt "a heavy load about his heart." When the vein was opened, the blood trickled slowly from the wound, but it soon came in a jet. By the time eight ounces were taken, the rigors ceased, and he expressed great surprise at the suddenness of the relief: when twelve ounces were abstracted, he was free from all complaint, and his skin had a comfortable moist feel. He enjoyed a good night; he had no return of the intermittent, and his recovery was rapid.

I had an opportunity of seeing this man daily for some months afterwards, and his constant tale was, that he had not felt so well, or so much of a man, since he went to Walcheren. The only remedies he took after the bleeding were laxatives and infusion of quassia.

Case II.—James Atkinson, aged thirty-three, had had repeated attacks of ague. Was seized with severe rigors when on the top of the Carlisle mail, travelling to Edinburgh. The paroxysm was evidently produced by exposure in bad weather, first to rain, and then to a keen frost, with wet clothes. When I visited him in hospital, he had laboured under the rigors for no less a period than twenty-six hours: in truth, it was the most severe cold stage I had ever seen in any country. He had severe pain in the head, back, and loins; oppression at præcordia. Warm drinks and stimulants, and hot applications, had been employed without benefit. The agitation of his body was so great, that it shook the very bedstead on which he lay, and threatened to terminate in convulsions. Tongue loaded, but moist; breathing hurried and laborious; pulse sixty-five, oppressed; skin not below the natural standard over the trunk, but all his extremities were cold, and he complained of a sensation of extreme cold. Fortunately I made a good orifice, which is not always easily done from the tremors, and the blood flowed in a better stream than I had ever seen it in the cold stage: twelve ounces were abstracted in three minutes, with very trifling relief, except to his breathing; but, during the flow of the second pound, he became more and more easy, and the rigor ceased completely. This pound was abstracted in two minutes: the arm was tied up on the approach of syncope, from which, however, he soon recovered. He lay quite easy; his body, and even the extremities, became of a proper temperature, and his skin felt moist; his pulse rose from 65 to 106; he passed a good night; had several stools during the next twenty-four hours: was found perfectly easy next day. On the following day he was convalescent, looked well, and asked for more food, and had no return of the disease.

The conclusions drawn by Dr. Mackintosh, from these and other cases, are as follow:—

1. I need scarcely say, that bleeding in the cold stage will not necessarily produce death.

2. That this practice will sometimes cure the disease; at others it will prove beneficial by breaking the chain of diseased action, and rendering the subsequent paroxysms milder and milder.

3. That bleeding in the cold stage, in every case in which it has been yet tried, has cut short the cold fits, and has prevented the subsequent stages of the paroxysm, so that the hot and sweating stages are saved. It seems to operate by anticipating the natural efforts of the constitution, removing the internal congestion, and restoring the lost balance of the circulating system.

4. That it promises to be most serviceable in severe autumnal intermittents, and more particularly in the pernicious and malignant fevers, as they are termed, of Italy, Holland, and other marshy countries, which are well known to be very fatal under the ordinary treatment. In these cases the reaction of the system cannot fully develope itself, in consequence of the extent to which internal congestion has taken place, and which this practice will remove.

5. That it may be used with safety in any climate where the cold stage continues long and threatens danger.

6. That bleeding in the cold stage is, at all events, more successful than in the hot stage, or than in the intervals. For although I have often seen bleeding used in such circumstances, and with advantage, by mitigating unpleasant symptoms, yet I have never known the subsequent paroxysm prevented by it.

7. The practice may be adopted in the first stage of all fevers; and probably will be found useful by surgeons in concussion of the brain.

8. If these cases possessed no practical merit whatever, they promise to be productive of great advantage to medical science, by destroying the very foundation of the erroneous system of Cullen. The doctrines upon which this system is founded have, to this day, bewildered old and young in the profession, who think and act only under the nod of authority. Cullen's system has been a great bar to all improvement in medicine; and is the principal cause of the backward state of pathology in this country, when compared with the strides made in that department by our professional brethren in France.

*Homœopathia; or the Art of Curing founded on Resemblances.*—It is not without some allowable degree of pride that the students of the British schools of medicine may look abroad into the occupations of the members of our profession in some other countries. The French are quarrelling most bitterly concerning the constant existence of what are called irritations, or those of them who are in better humour are pursuing the visions of animal magnetism. The Italians have not yet quite made up their minds concerning the infallibility of the system of John Brown. The Germans are occupied in matters of higher importance, in nursing with all the care and industry which belongs to their character the two very useful sciences of *animal magnetism* and *homœopathia*. In this country we do not pursue these matters very far; believing, for the most part, that ingenuity and philosophy are very good things, but that a little good sense is a very useful quality also.

We have on former occasions laid before our readers perhaps even more particulars than they read concerning Broussais and his disciples, and concerning the resurrection of magnetism. The science of homœopathia was, we think, merely incidentally alluded to, some time ago, in connexion with the asserted power of belladonna to prevent scarlatina, by its property of producing a state something like it. If the reader, therefore, has nothing useful to do for a quarter of an hour, and feels interested in watching the imaginative excursions of physicians out of the ordinary paths of practice, a short explanation of this famous subject may be worth his perusal.

The term homœopathia is derived, we believe, from ὁμοιον παθος, a *similar affection*, and the system, which it is intended to convey an idea of, has risen up within the last twenty years: at least, so we learn from the foreign journals, although it seems to us to be a kind of revival of old fancies, or the celebrated doctrine of signatures in a new shape. The great principle of it is, *similia similibus curantur:* and, to obtain a complete cure, it is only necessary to obtain some article of the materia medica which possesses the property of producing an artificial malady with as close a resemblance as possible to that which you wish to remove. Two diseases, say the founders of this doctrine, cannot exist together; and the *medicamental* disease, being stronger than the *natural* disease, ejects it altogether from the body. And, if it is suggested that the human body, in that case, has only got rid of a weak disorder by admitting a stronger,—has been put out of fear of an enemy by letting in a master,—we are told that the medicamental malady always cures itself. A few exceptions to the above doctrines seem to be admitted, however, such as small-pox, syphilis, scabies, and the plague; which, with some others, are allowed to be too much for the medicamental malady. But in other cases the medicamental virtue is so absolute, that the smallest possible dose is sufficient to dislodge the disease; and as it immediately, of course, produces a change in the condition of the human body, the same medicine is never to be given twice. If the patient begins to mend, you must give nothing; if he is getting worse, the medicament was not well chosen: you may be inclined to suppose that it might do better in a larger dose, but that is not allowed. If the patient is neither better nor worse for the medicine, the conclusion is, not that you have given *too little* of it, but *too much*. Thus the ordinary doses of the homœopathic physicians is about the *millionth* part of a grain, or not quite so much. Decoctions, infusions, and extracts, seem to be very little employed by them, but only powders and tinctures. They are very severe in the articles of diet and regimen, a severity to which all systems of quackery are more or less indebted.

The reader is not to suppose we have no cases to present to him. A brazier's apprentice was troubled with a quartan fever for three months, which did not yield to strong doses of kina. He came under the care of an homœopathic: *a drop* of the tincture of bark was given him, and even repeated, but without success; and his physician remarking that he had a troublesome cough which prevented his sleeping, had recourse to the hyoscyamus, and gave him the *millionth part of a grain*. The cough and the fever disappeared together.

An emaciated, feeble woman was suddenly attacked with hemorrhage from the nose, the lungs, and the stomach: cinchona, canella, chalybeates, applications of vinegar, were tried in vain; and the patient was left to her fate. An homœopathic physician very providentially saw her, the hemorrhage still continuing, and the patient cold and almost insensible: a *billionth* of the powder of chamomile was given without delay, and the hemorrhage ceased in an instant. The patient slept remarkably well, but felt a little low the next morning. A new attack of epistaxis came on: the condition of the body was of course changed; the powder of chamomile no longer proper; but a *billionth* of a grain of saffron was given, and in a quarter of an hour all was well.

The same patient was attacked with the ague a few days afterwards, and the same enlightened physician who had conducted her safely through her former difficulties now tied a *leaf* of the Flammula Jovis to her little finger, which produced a little blister, and the fever was cast out in the most summary manner. The Flammula Jovis is, it seems, very commonly employed in this manner, and might surely lead to the saving of a great deal of bark in the fens of Lincoln and Cambridge.

One more case:—A robust man, of thirty-eight, affected for *six years* with gonorrhœa, which had been vainly tried by various practitioners according to the common methods, placed himself under the care of an homœopathic physician. The discharge was yellowish and copious; there were often painful erections, but there was no pain in making water; sometimes there was much pruritis at the extremity of the glans. The physician, *having remarked* that *analogous* symptoms were produced by the fresh juice of *parsley*, caused the patient to take, in the morning, *one drop* of this juice in spirits, mixed with half an ounce of water, enjoining strict abstinence, and desiring that no other medicine should be given. The next day the discharge was greater: this merely shewed the combat going on between the natural and medicamental disease; and, although not strictly, as it appears to us, in conformity to the principles of homœopathia, the same medicine was given, in the same dose, the second day; and on the third day the patient was well.

We have no more cases before us at present; but the list of diseases to which the new, or, as we think it may not unaptly be called, the *Lilliputian* system of medicine has been successfully applied, would take up a page of our Journal. We observe among them phthisis, acute rheumatism, madness, and *sterility*.

It is quite unnecessary for us to point out the great importance of the new system as regards the practice of physic in general, or its vital importance as regards the Apothecaries' Company in particular. The fullest exposition of it seems to be contained in a work by Dr. Boeckel, published recently at Strasbourg. Several other publications on the same subject are mentioned in the Bulletin des Sciences Médicales for January last. (*London Medical Repository*, May.)

*Case of Recovery from the Narcotic Effects of Opium, by means of Artificial Respiration.* By HUGH J. OGILVIE, M.D. of Madison, Morgan County, Georgia. (From the North American Med. and Surg. Journal.)

On the 26th of September last, I was in attendance on Mrs. R—, who had been delivered, ten days prior to my visit, of a strong and healthy child. From the rude and officious interference of the midwife at the time of delivery, considerable injury, or abrasion of the meatus urinarius, had ensued; attended with retention of urine, and extensive inflammation, towards the termination of her convalescence. I prescribed an anodyne at bed-time, consisting of twenty-five or thirty drops of laudanum: through mistake, the dose was administered to the infant, whom I found in the following condition, on repeating my visit early the ensuing morning:—Face of a dark, livid colour; respiration slow and oppressed, repeated about six or eight times in a minute; pulse slow, weak, and tremulous; extremities cold; coma, and dilated pupils. I was informed that it had had several convulsions a few hours after receiving the narcotic draught. I inquired if any thing had been administered, and was informed that it could not swallow. Not relying explicitly upon the assertion, I directed an infusion of coffee, and, on attempting its administration, found the power of deglutition suspended.

Viewing the case as desperate, and beyond the ordinary resources of the profession, it occurred to me, from the peculiar state of respiration, that, if that function could be sustained, the life of the infant might be saved. Accordingly, a female attendant was requested to close the nostrils, and blow into its mouth, and press upon the abdominal muscles and diaphragm, so as to carry on artificial respiration. This was persevered in for half an hour without much apparent amendment, except an occasional convulsive effort to expire the air blown in. Perseverance was enjoined, and in two or three hours I was informed that respiration was completely re-established: the face assumed its natural colour, and all the functions were restored to their normal condition. No unpleasant symptoms ensued, and the child is now in the enjoyment of perfect health.

*Reflections.*—I was induced to adopt the practice instituted in the above case, from having noticed, in a periodical work, a report of a similar case by a physician in Boston, whose name I have forgotten. The case alluded to was unsuccessful, but the theory which suggested the remedy was in perfect accordance with my own views. Opium in large quantities operates primarily upon the nervous system, and is mainly expended upon the respiratory and sympathetic ganglia. It therefore follows, as a necessary corollary, that, if respiration can be sustained by artificial means till the sedative influence of the opium has been subdued by the recuperative energies of the system, life may be restored. If my opinion of the *modus operandi* of opium be unscientific or erroneous, the practical deduction may nevertheless be of service. These views, however imperfect, I hope may be the means of attracting the genius and talent of some one more adequate to its elucidation. Should a more extensive trial of the remedy be attended with success, we have another auxiliary that will tend to lessen the *opprobria medicorum*.

### SURGERY.

*Cases of Hemorrhage from the Urethra, with Remarks.* By GEORGE F. LEHMAN, M.D. (From the Philadelphia Journal.)

Hemorrhagy is at all times alarming to the individual affected, and not seldom to the professional attendant.

The extreme anxiety and apprehension created by a ruptured blood-vessel, could not escape the notice of the most superficial observer, and the means for its suppression ought to be prompt and effectual.

If it occurs in consequence of previous disease, the surprise is not so great as when it happens in the midst of robust health, and from parts of the body particularly guarded against accidents, and protected with fastidious care.

From the inflammation of gonorrhœa extending along the urethra, the mucous membrane of the bladder sometimes becomes ulcerated, and an effusion of blood is the consequence.

An imprudent use of strong astringent injections to check gonorrhœa will have a similar effect, especially when no regard is paid to moderate habits by the patient, but he lives as usual on high seasoned food, and partakes freely of wines and spirituous liquors.

It occasionally, but rarely, happens that the bleeding is so profuse after the application of caustic for the removal of stricture in the urethra, that the patient faints; but this is the extent of the evil: death never follows such hemorrhagy.

Moderate discharges of blood from the penis happen now and then from other causes, but generally symptomatic of disease of the parts.

In all casualties of this kind, the most approved practice consists in administering sedative and astringent injections of sugar of lead and sulphate of zinc in rose-water, and solutions of alum, and pledgets dipped in the same to be applied externally, enjoining perfect rest, with internal astringents, and cold applications to be used according to the condition; and, if these fail, pressure to the perineum.

Hemorrhagy from the penis, however, I believe is not very common under any circumstances; and, as three cases have fallen under my observation, unaccompanied by other affections, I take the liberty to present them to the medical public.

Case I.—Joseph Frendly was employed as a temporary nurse in the Lazaretto Hospital, September 1817. At that time the hospital was crowded with mitior and gravior cases of typhus fever. He was a short but stout-built man, forty years of age.

About three o'clock in the morning of September 15th, 1817, I was called up to see him. The messenger declared he was bleeding to death, and had fainted. I went to the hospital with all possible speed. The blood was running freely from his urethra on my arrival. I presume from appearances he may have lost from twenty-five to thirty ounces. He could not tell when it had commenced. It originated during his sleep, and he was aroused from his slumbers by a sensation something of the nature of nightmare. His pulse was seventy-nine per minute. I applied ice immediately to the perineum and scrotum, and in four minutes the bleeding ceased. I advised him to keep to his bed, and administered one ounce of Epsom salt. By eight o'clock he had two fecal evacuations. At night the hemorrhage returned, in consequence of his walking through the room. Ice was again applied as before, and the bleeding stopped in three minutes. I now directed him not to leave his bed without my permission. For several days his urine was slightly discoloured with blood. He was confined to a diet of oatmeal-gruel, and at rest, and in five days he was well. At the period of his attack, this man was in perfect health. Eighteen years preceding this event, he suffered a slight attack of gonorrhœa.

Case II.—James M'Cauley, aged fifty-one years, was carpenter of the quarantine station, and acted as bargeman during the summers of 1820, 21, and 22. He was a man of temperate habits in general, and had been married many years.

On the 31st of July, 1822, he called on me early in the morning, in considerable agitation, and stated that, on awakening about daylight, his penis was erect and painful, and blood was rapidly flowing from it. He supposed a pint of blood was lost. At the time of this incident he was in a dream of coition with his wife. He had no sexual intercourse for two months preceding, and declared himself very moderate in the love of women. The rigidity of his penis was unnatural, and created uneasiness, but he felt no pain from the discharge of blood. He had not been engaged in any hard work, nor made any heavy lift; but was naturally of a costive habit, and would remain six and seven days without any alvine discharge. At this time, five days had elapsed without a fecal excretion, and he strained very hard at stool. He never had the venereal disease. In 1811, in consequence of a fall, three of his ribs were broken on the left side.

I gave him an ounce of Castor-oil, which operated twice. The penis and scrotum were repeatedly bathed in cold water from the well. During the whole day blood was emitted by drops, and at night about a wine-glassful.

August 1st.—Blood continued to dribble from his urethra, and he complained of a burning sensation when he urinated. I put him on the antiphlogistic regimen, and flaxseed tea as his only drink.

2d —Discharged from ten to twelve drops of blood every hour. Application of cold water continued. I intended to use an injection of Sulphate of Zinc, but he objected; and I prescribed Sugar of Lead, three grains morning and evening, flaxseed tea, and low diet. He complained of ardor urinæ for two days, and a slight pain in the lower part of his abdomen until August 2d.

3d.—Bowels not open since July 31st. Administered an ounce of Castor-oil, which had the desired effect, and operated five times.

My case-book concludes thus:—He continued taking the sugar of lead, drinking flaxseed tea, living low, and at rest, until August 6th, when all traces of the hemorrhage had disappeared.

Case III.—Mr. F. W. aged seventy-six years, consulted me May 17th, 1825. He had a regular dropping of blood for six days from the urethra. When he called on me, pulse sixty-nine in a minute. I administered an ounce of Castor-oil, and advised him to bathe the penis in cold water repeatedly. He returned in two days, and informed me that the bleeding had increased. Apprehending, from the age of my patient, that his might be a case of atonic hemorrhage, I prescribed injections into the urethra, composed of one drachm of Alum to eight ounces of water, a table-spoonful three or four times a-day, and fifteen grains of Kino to be taken morning and evening. In four days the bleeding ceased, and he has experienced no return of the complaint.

---

### CHEMISTRY.

Dr. Granville *on the Disinfecting Liquid of Soda.*—M. Labarraque, a pharmacien residing in Paris, proposed, two or three years ago, to employ chlorine in a liquid form, in lieu of Morveau's method hitherto adopted, for disinfecting air in which putrid animal effluvia are disseminated, and for arresting putrefaction in dead bodies. For this purpose he selected, first, a solution of the salt formerly termed oxymuriate of lime in water; and secondly, a solution of carbonate of soda saturated with chlorine gas.

To these liquids M. Labarraque gave the names of Chlorure d'Oxide de Calcium, et d'Oxide de Sodium, corresponding to our chlorides of lime and soda; and under these denominations he promulgated, with a becoming spirit of liberality, his discovery of their disinfecting properties. The promulgation, however, was not, as one had a right to expect, accompanied by any scientific inquiry into the real constitution of the liquids, nor by any analysis of their ingredients. Nor was it followed by any attempt to explain the curious and important facts which they had brought to light, and which were in a short time confirmed by the observations of many. Labarraque's views were formed, in Dr. G.'s opinion, on mere assumption, and were adopted by the French, and by all the English translators and commentators who assisted in making his discovery more generally known. No one seemed to doubt the correctness of those views, until Dr. Granville, having had occasion to use very extensively one of the liquids in question, (that containing soda,) undertook the analysis of that liquid, in order to ascertain its real composition; and he entered upon an inquiry into the phenomena resulting from the action of chlorine on animal matter in a putrid state. The results of these inquiries Dr. Granville has detailed at full length in his paper, in which a series of experiments is minutely described, from which the author conceives he has proved that the supposed "chloride of *oxide* of sodium" in solution is in reality a mixture of—

| | |
|---|---|
| 73.53 | dry chloride of sodium |
| 26.47 | neutral chlorate of soda |
| 100.00 | |

with an excess of chlorine equal to twice the bulk of the water employed in preparing the liquid agreeably to Labarraque's own formula.

Besides detailing the several analytical and synthetical experiments in support of the above conclusions, Dr. Granville attempts to prove the accuracy of those conclusions by the application of the atomic doctrine, as well as by a calculation of the weight and measure of the chlorine gas required to form the disinfecting liquid: whence it appears that, during the process of preparing that liquid, five atoms of oxygen combine with one atom of chlorine to form chloric acid, which unites with one atom of soda to form the chlorate of soda; and five atoms of sodium combine with five atoms of chlorine to form five integrant atoms of chloride of sodium. These combinations are not due to the decomposition of water, but to a peculiar arrangement of the elements contained in the solution. With regard to the quantity of chlorine gas employed, it appears to be very considerable. Twenty fluid ounces of the liquid contain 503.36 cubic inches of that gas, (besides the free chlorine,) which weigh, according to Thomson's tables, 383.815 grains; and as the soda dissolved in those twenty ounces of liquid weighs 341.185 grains, according to the atomic theory, the weight of the solid contents of that quantity of liquid ought to amount to 725 grains; which is precisely what Dr. Granville found on evaporating the whole of the liquid to dryness. If this be the real composition of Labarraque's liquid, it is clear that no such compound as the chloride of oxide of sodium exists in it, and its present denomination must be incorrect: Dr. Granville therefore recommends that it should be abolished, and that the simple name of Disinfecting Liquid of Soda should be substituted for it.

In another part of his paper, the author endeavours to show that the singular properties of the above liquid are due entirely to the chlorine, and in no way to the agency of the salts contained in it. The same results are obtained when a simple solution of chlorine water is employed; but in that case the escape of the gas is considerable, and consequently offensive to the operator and assistants. This is not the case when the same quantity of chlorine is thrown into a solution of the two salts mentioned in the course of the paper: so that Dr. Granville infers that the presence of those salts serves to lessen the tendency of the free chlorine to escape in a gaseous form. When the two salts, alone obtained by evaporation, are redissolved in distilled water, no disinfecting effects are obtained, although so large a proportion of the chlorine enters into their composition; but if two measures of that gas, equal to twice the bulk of the solution, be thrown in, all the disinfecting properties are restored to the liquid.

Dr. Granville promises to lay before the Royal Society a continuation of his inquiries on this subject, in which he will describe the mode of action of the disinfecting liquid on putrid animal matter,—detail the new compounds that result from that action,—point out a method of ascertaining the presence of animal effluvia in the air by means of chlorine, applicable in times of infectious diseases,—and, lastly, suggest a more easy and economical process of preparing the disinfecting liquid. (*Philosophical Magazine.*)

*On the Chlorides of Lime and Soda.* By R. Phillips, F.R.S. L. & E. &c.—These chlorides have lately excited considerable notice, owing to the recommendation of M. Labarraque; and not only in France, but in this country also. Should they be found to possess a moderate share only of the powers which have been attributed to them, they are exceedingly important compounds, and their exact nature, as well as their mode of action, is entitled to a more careful examination than, as appears to me, they have hitherto received.

The chloride of lime has long been known under the name of oxymuriate of lime, or bleaching powder: it is prepared, as is well known, by passing chlorine gas over hydrate of lime; the resulting compound, put into water, yields the chloride of lime in question; it is also sometimes formed by passing the gas into water containing lime in suspension. This chloride is now proposed to be employed, and it appears with great success, as a disinfecting substance.

The existence of such a compound as chloride of soda, or potash, has hitherto not been so clearly ascertained as that of the chloride of lime. Two modes of preparing this compound have been proposed, one by M. Labarraque, and the other by M. Payen: the former passes chlorine gas into a solution of carbonate of soda, while the latter proposes to decompose chloride of lime by carbonate of soda.

I have tried both methods, and both are very easy of execution. When chlorine gas is passed into the solution of carbonate of soda, it is very readily absorbed, and this absorption takes place without expelling a particle of carbonic acid: the solution has a faint smell of chlorine: when heated, scarcely any chlorine is evolved, and the solution first acts as an alkali upon turmeric paper, and then bleaches it: on the addition of an acid, chlorine and carbonic acid gases are evolved.

When evaporated until a slight pellicle appears, a mass of fibrous crystals is soon formed, the consistence of which is almost pulpy, owing to the retention of the solution by the capillary attraction of the crystals. When these crystals have been separated, the solution yields minute crystals of carbonate of soda, possessing the usual form of that substance.

These filamentous crystals are too minute to admit of any examination of their form: they appear to me to consist of chlorine, carbonic acid, and soda, or chlorine in combination with carbonate of soda. When put into a solution of indigo in sulphuric acid, they immediately decolorise it, by the evolution of chlorine, accompanied with carbonic acid. I have not yet had an opportunity of analysing this compound: I find, however, that, whilst drying by exposure to the air, it loses so much chlorine (I presume by the action of carbonic acid,) that it does not contain two per cent. of it in any state, either of mixture or combination. I have not particularly examined the solution formed by decomposing chloride of lime by means of carbonate of soda; but I find that it retains its bleaching power after ebullition, and yields crystals by evaporation.

In the last Number of the Phil. Mag. and Annals, an account was given of a paper of Dr. Granville's, read before the Royal Society, on the Composition and Action of the Chloride of Soda. According to Dr. Granville, the disinfecting properties of chloride of soda are entirely dependent upon the uncombined chlorine gas which the water holds in solution. Now, admitting for a moment this to be the case, and that no such compound as chloride of soda exists, the same explanation cannot apply to the action of the chloride of lime: and it is singular that Dr. Granville has taken no notice of this substance, although, according to M. Labarraque, it is generally employed for disinfecting apartments; while the chloride of soda "is especially employed in topical and external applications to wounds and ulcers affected with hospital gangrenes, &c." (Alcock on the Use of the Chlorurets, p. 126.)

Some late experiments have proved in a most decided manner that the explanation which Dr. Granville has offered, is not a correct view of what actually occurs. M. Gaultier de Claubry has shown that air which has passed through putrid blood, and afterwards into a solution of chloride of lime, was rendered inodorous, and was completely purified, occasioning the precipitates of carbonate of lime; but, in a similar experiment, the fetid air was passed through a saturated solution of caustic potash: the chloride of lime had then no effect upon it, and it retained its insupportable odour. This is decisive as to the action of the carbonic acid of foul air in evolving the chlorine by which it is purified.

I have already mentioned that chloride of soda does not, even by ebullition, lose its bleaching property; and this is another proof that its action does not depend upon the mere gas which it holds in solution; for it will hardly be maintained that any circumstance less than combination will retain chlorine in water at a boiling temperature. It also retains its power to a considerable extent even after evaporation to dryness.

Dr. Granville states that the salt in question is a mixture of 73.53 of chloride of sodium and 28.47 of chlorate of soda. I am quite at a loss to conjecture how Dr. Granville obtained this result, either by calculation or experiment. For preparing the chloride of soda, M. Labarraque directs that a solution of 288 parts of crystallised carbonate of soda is to have the chlorine evolved from the decomposition of 66 parts of common salt passed into it.

Now, as 288 are equivalent to 2 atoms of crystallised carbonate of soda, there will be required the chlorine of 2 atoms = 120 of common salt to convert them into chloride of sodium and chlorate of soda; and even admitting, what I believe is not the case, that the chlorine of the 66 parts of the common salt converts, as far as it goes, the carbonate of soda into chlorate of soda and chloride of sodium, the quantity is so deficient that the dry salt must consist very nearly of Chloride of Sodium, 45, Chlorate of Soda, 16; Carbonate of Soda, 39; = 100. (*Ibid.* No. 5.)

